# CANoE: A Context-Aware Notification Model to Support the Care of Older Adults in a Nursing Home

**DOI:** 10.3390/s120911477

**Published:** 2012-08-24

**Authors:** Sandra Nava-Muñoz, Alberto L. Morán

**Affiliations:** 1 Faculty of Engineering, Universidad Autónoma de Baja California, Km 103 Carretera, Tijuana-Ensenada, Ensenada, B.C. 022860, Mexico; 2 Faculty of Engineering, Universidad Autónoma de San Luis Potosí, Av. Manuel Nava #8, San Luis Potosí, S.L.P. 078290, Mexico; 3 Faculty of Science, Universidad Autónoma de Baja California, Km 103 Carretera, Tijuana-Ensenada, Ensenada, B.C. 022860, Mexico; E-Mail: alberto.moran@uabc.edu.mx

**Keywords:** context-awareness, notification, healthcare in a nursing home, monitoring, coordination

## Abstract

Taking care of elders in a nursing home is not an easy task. Caregivers face two major problems: a lack of awareness of the situations surrounding the elderly care and the lack of information regarding the availability and the activities of other caregivers to support their coordination process. Various efforts have proposed solutions to cope with these problems, but they do it without considering all the requirements imposed by the criticality of this type of environment. In this paper we propose CANoE, a model for the design of context-aware notifications in critical environments, such as a nursing home. The main feature of this model is that it considers three sources of context (the environment, and the issuer and the receiver of the notification) for adapting the content, the terms of delivery and the presentation of the notification message. Based on the CANoE model we developed the CANoE-Aw and CU-IDA systems, which were evaluated through two case studies in a nursing home. The results of these evaluations provide evidence that caregivers achieved an increased awareness of the situations of care of the elderly and perceived the systems as adequate tools to support their coordination while attending a situation of care.

## 1. Introduction

Caring for an older adult may be complex; this complexity arises from factors such as the elder's physical and mental health status, the degree of independence in the execution of his/her activities, *etc.* [1]. According to the latest estimates of the United Nations, the worldwide elderly population (65 years and over) is expected to grow [2]. This increase in population affects the number of older adults with disabilities. Data from the World Health Organization indicate that 7.05% of the World population are older adults with moderate or severe disability [3].

Researchers from the Ambient Intelligence (AmI [4]) community have proposed applications to assist the elderly in the development of their activities of daily living [5–7]. However, when such assistance is no longer effective, a higher level of support is required. This support may come from some kind of personalized caregiver (formal, informal or professional [8]), and sometimes from an institution specializing in the care of older adults with certain illnesses. An example of this are nursing homes or day care centers, which are public or private institutions engaged in the coordinated care of people [9].

Application development in nursing homes differs from the development for other environments such as hospitals, homes or offices. The main difference lies in the criticality of the environment in terms of: the types of events/situations that arise (e.g., accidents of the elderly [10]), staff (caregivers) and their mobility within the environment (e.g., high mobility of caregivers due to the various activities that they carry out [11]) and the (care) activities performed by this staff (e.g., residential caregivers are responsible for monitoring and caring for older adults [12]). Therefore, nursing homes can be considered as a critical environment where the wellness of the elderly is the main motivation of the care process.

However, some of the risks identified in the elderly care process in this environment include situations that arise due to the physical and mental decline of the patients, including aggressions, disorientation, wandering, and falls, among others [13]. As part of their care duties, caregivers perform multiple activities at different locations within the residence. For this reason, they are unable to stand beside all seniors at all times. Thus, caregivers are faced with a diversity of problems, including: (i) a lack of awareness on the status of each elder, particularly when faced with critical situations; and (ii) a lack of strategies to support communication between caregivers when they need to coordinate their efforts to cope with a care situation of the elderly.

Faced with these problems, the need for mechanisms that provide support for caregivers in their care activities becomes evident. In this work we propose the use of context-aware notification mechanisms that allow the caregiver, on the one hand, to become aware of the different situations of care of the elderly, and on the other hand, to coordinate their work to provide a better care service for the elderly under their care.

The literature reports individual efforts either to notify the caregivers in nursing homes about a situation of risk [10,14,15], or to support coordination among members of a network of caregivers [16,17]. However, to the best of our knowledge, there are no notification schemes that provide general solutions to these problems in nursing homes; that is, there are no tools that could serve as specialized foundations for the design or construction of context-aware notifications in this kind of critical environments. In this sense, we have identified some context-aware applications that consider adapting notifications at design time, in a *passive* manner. This means that they analyze the notification receiver's profile and then design a single mechanism to display the notifications, and consider by default that the notification's presentation is performed in an adequate manner [6,8]. Also, others works [18,19] have considered to notify through several mechanisms in an *active* manner. This means that they select in real time the notification mechanism to be used; however the adaptation is made only considering the context of the issuer of the event (*i.e.*, they do not consider the receiver's context). Thus, these works do not consider the physical availability of the receiver of the notification in order to adapt its presentation.

The aim of this paper is to propose a model for the design and development of context-aware notification systems, which take into account the requirements of a critical environment (as the physical availability of the receiver of the notification). For this type of environment it is essential that the notification receiver not only receives the information on time, but also that s/he perceives and understands it in order to timely act on the critical situation that generated the event.

To validate the model, we propose the development of two notification systems, with which to address the two issues identified in the process of care for the elderly in a nursing home. The focus of our proposal regards to context-aware systems, which allow for the generation of Ambient Intelligence (AmI) which provide a proactive and sensitive solution to the needs of users [20].

The rest of the paper is organized as follows: Section 2 describes the proposed model, and defines each of the design guidelines for CANoE notifications. Later, Section 3 describes a basic monitoring system to obtain information from care situations in the residence. Sections 4 and 5 present two case studies about two notification systems created based on the CANoE notification model (CANoE-Aw and CU-IDA), CANoE-Aw focuses on the lack of awareness issue of caregivers during their care activities, and CU-IDA focuses on the lack of information issue for caregiver coordination while attending a situation of care. Finally Section 6 concludes our work by describing our main findings, as well as our directions for future work.

## 2. A Model for Context-Aware Notifications for Critical Environments (CANoE)

This paper proposes the concept of *C*ontext-*A*ware *No*tifications for critical *E*nvironments (*CANoE*) as a notification mechanism that takes context into consideration to adapt the delivery, content and presentation of a notification. CANoE is designed to be used in critical environments by taking into consideration three sources of context for adaptation: (i) the recipient of the notification; (ii) the issuer of the notification; and (iii) the characteristics of the environment where the notification occurs.

This type of notification is proposed for critical environments such as nursing homes, where the emergence of diverse situations may affect the physical integrity of the elderly; which we refer to as *Situations of Care* [12].

**Figure 1.**
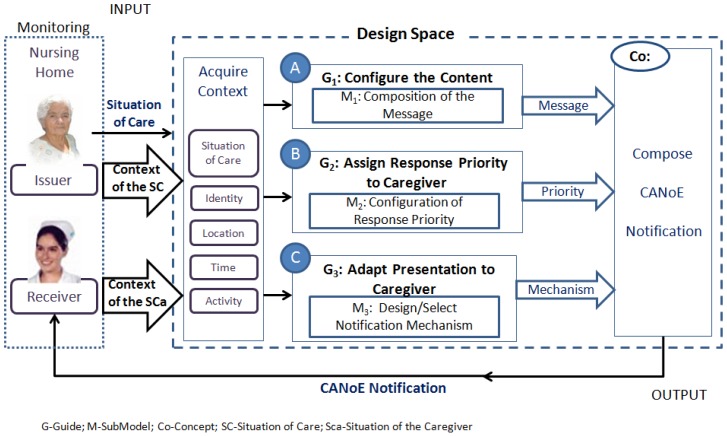
Schematic view of the CANoE (Context-Aware Notification for critical Environments) model for a nursing home.

The proposed CANoE notification model considers contextual information about two actors: (i) the elder with cognitive impairment, as the person being cared and about whom the event is generated (issuer); and (ii) the caregiver and his/her environment, as the person who provides care and who receives the notification (receiver). The model includes three design guidelines and three sub-models that operationalize each of these guidelines. Figure 1 shows a schematic view of the CANoE notification model, which considers as inputs the actual situation of care, the context of the elderly involved in the situation of care, and the context of the situation of the caregivers. The latter two include the environmental context features of the locations of caregivers and older adults. The model adapts the content (A), the delivery (B) and determines the presentation mechanism (C) based on the context through the following three design guidelines: Configure the Notification Content (G1), Assign Response Priorities to the Caregiver as the receiver of the notification (G2), and Adapt the presentation of the notification to the caregiver (G3). Finally, the outputs of each of these adaptation processes (Message, Priority and Mechanism) are integrated to form a CANoE notification.

**Figure 2.**
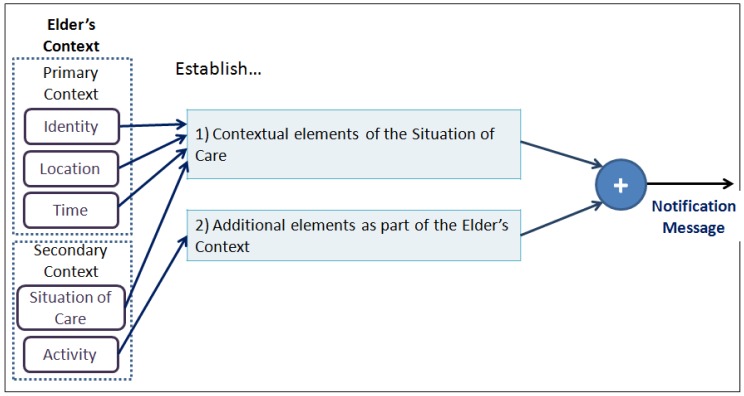
Model for the Composition of the Notification Message.

**Figure 3.**
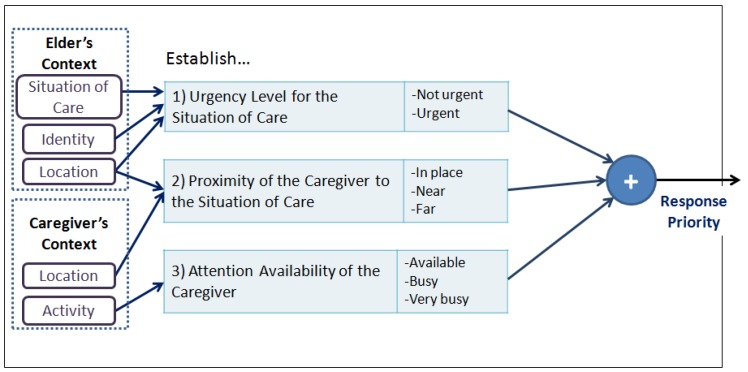
Model for the Configuration of the Caregiver's Response Priority.

**Figure 4.**
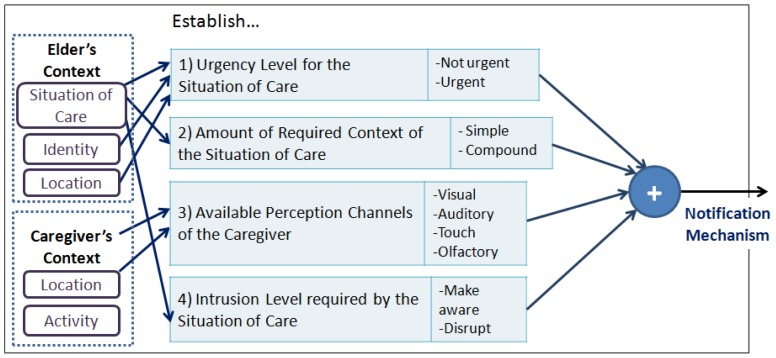
Model for the Design and Selection of the notification mechanisms.

Details about the three design guidelines that should be considered for the construction of a CANoE notification follows:

- *Configure the contents of the notification according to the context of the Situation of Care.* This guideline proposes to adapt the content of the notification considering the question *What to notify?*, and considers the ‘Composition of the notification message’ sub-model depicted in Figure 2. To configure the contents of the notification we should firstly establish the contextual elements that describe the situation of care to be notified (e.g., identity, location, *etc.*); and secondly, the contextual elements of the elderly that enrich the notification message (e.g., activity). This in order to allow the caregiver to understand what is happening regarding the event (situation of care) and based on this understanding to take appropriate measures to provide care for the elderly.
- *Assign response priorities to caregivers.* This guideline proposes the adaptation of *When to Notify?* and *Who is notified?* and presupposes the existence of more than one caregiver in the care process. This notification seeks to support coordination among caregivers by providing a *response priority* to each of them. This priority indicates the caregiver's availability to address the reported event in comparison to the other caregivers.
  The caregiver who receives the highest response priority is the person whose status shows the highest availability, in terms of their activity and location, to address the situation of care reported. For this reason, three priority levels are proposed: High, Medium and Low. To configure the response priority the ‘Configuration of the Response Priority’ sub-model is used (see Figure 3). This model is a function of the following three input parameters:
  - *Urgency Level required by the situation of care*, refers to the urgency level with which the situation of care should be dealt with. This urgency level is derived from the context of the older adult and his/her environment. The values that it can take are: *Non-immediate* or *Immediate*.
  - *Proximity of the caregiver to the location of the event*, refers to the distance between the location of the caregiver and the location where the situation of care is taking place. The values it can take are: *Same place, Near* or *Far*.
  - *Caregiver's Attention Availability*, refers to the availability of the caregiver to address the notified event. This availability is a function of the criticality level of the caregiver's current activity, which is obtained based on the caregiver's context; the higher the criticality level of the activity, the lower the caregiver's availability. The values that it can take are: *Available, Busy*, or *Very Busy*.
  Based on these three values we obtain the result of the response priority of a caregiver. As an example, let us consider a situation of care that has a level of required attention equal to ‘Immediate’, and the presence of two caregivers (C1 and C2) with the following states: C1 (‘Near’, ‘Available’) and C2 (‘Near’, ‘Very Busy’). In this case, the configuration model should assign a ‘High’ priority response to C1 and a ‘Medium’ priority response to C2.
  The main motivation of this guide is to assist in the coordination of caregivers to address the situation of care reported; it is assumed that if the caregiver knows his/her priority of response, s/he may make informed decisions about: (i) addressing the notified situation of care; or (ii) continue performing the activity s/he was doing at the time of the notification.
- *Adapt the presentation to the caregiver*. This guide proposes the adaptation of *How?* and *Where to report?*. For this, we should consider the creation of different notification mechanisms with the aim of using them under the conditions that arise at the time of notification. The model that addresses this guide has two main purposes: (i) supporting the design of CANoE notification mechanisms; and (ii) supporting the decision to select the mechanism by which to notify. Figure 4 shows the model of the ‘Design and Selection of the notification mechanism’, which comprises:
  - *Urgency level required by the situation of care*, similar to the previous model.
  - *Amount of context required from the situation of care*, refers to the amount of contextual elements that the notification mechanism will be able to represent. This dimension is a function of the number of elements in the context of the situation of care that are necessary to enable an understanding of the notification. The values it can take are: *Simple* (one contextual element) and *Compound* (two or more elements).
  - *Available perception channels of the caregiver*, refers to the caregiver's available senses to attend a notification when a situation of care is notified. This is obtained from the activity performed by the caregiver and the characteristics of its location within the nursing home environment. The values it can take are: *Visual, Auditory, Tactile* and *Olfactory* (in conjunction with the sense of taste).
  - *Intrusiveness level required by the situation of care*, refers to the level of attention to be obtained from the caregiver in order to receive the notification. The values it can take are: *Make aware*, or *Disrupt*.
  Based on these four values we determine the mechanism by which we should notify. As an example let us consider that there is a caregiver that has the *auditory channel* available, and that there is a situation of care, with an *urgent* urgency level, a *simple* amount of required context, and a *disrupt* intrusiveness level, then an appropriate mechanism to send the notification should provide an auditory notification and use an auditory clue to obtain the caregiver's attention.
  As shown in Figure 1, each sub-model generates an element of the notification (Message, Priority, Mechanism), which has been adapted *a priori* based on the acquired context. This way, a CANoE notification is formed. In the next section we describe a basic infrastructure for context acquisition in the nursing home. Later, we present and discuss two case studies that illustrate the applicability of the CANoE model.

## 3. An Approach to a Monitoring System for the Nursing Home

With regard to monitoring elders and caregivers in a healthcare environment, the literature identifies monitoring systems ranging from applications that: (i) seek to maintain the independence of the elderly in his/her daily activities through the use of temperature sensors [21] and motion sensors [22,23]; (ii) to applications that identify a risk for the dependent elderly or activities for the caregivers using temperature sensors [24], weight sensors [25], and computer vision [26,27]. As in other context-aware systems, firstly we capture the primary context (e.g., the *identity* or *location* of a person) and then based on it infer the secondary context (e.g., the *activity* of a person or the occurrence of a *Situation of Care*). Diverse works report high effectiveness indexes in their evaluations (e.g., 99.96% regarding inferred location [28], and 82.8% regarding inferred activity [29]).

In this work, as a proof of concept and to demonstrate the feasibility of monitoring the elderly at the nursing home, we designed and developed a basic infrastructure for the identification of three situations of care: Entering into sensitive areas, Escape from the house and Get up without assistance. This effort allowed us to illustrate the feasibility and reliability of sensing the data of both actors, which remains a key challenge.

**Figure 5.**
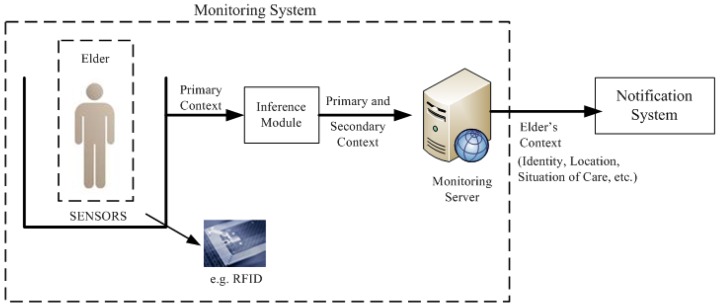
Architecture of the proposed Monitoring System.

Figure 5 shows the architecture of the proposed monitoring system. It considers a set of sensors around the elderly (e.g., the elderly carry RFID tags on their shoes and their jackets) to capture the primary context. Subsequently, this data is sent to an inference module to obtain the secondary context through various inference techniques (e.g., Decision trees [30], Bayesian networks [31], Markov models [5]) and finally this context (primary and secondary) is sent to a monitoring server, which sends the information to the notification system. Communication between the monitoring server and the notification system is achieved through the XMPP protocol using the OpenFire software [32]. This message protocol and format allows for the sending of the context of each event.

**Figure 6.**
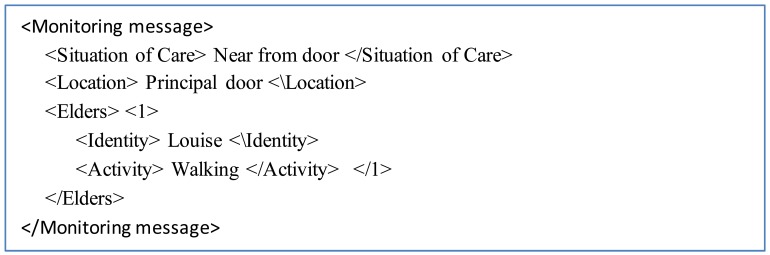
Format of a monitoring message.

Figure 6 shows an example of such an event message regarding a situation of care. The message follows the following format: situation of care, location, number of the elderly identified (involved in the situation of care), name of the elderly involved, and the inferred activity.

**Figure 7.**
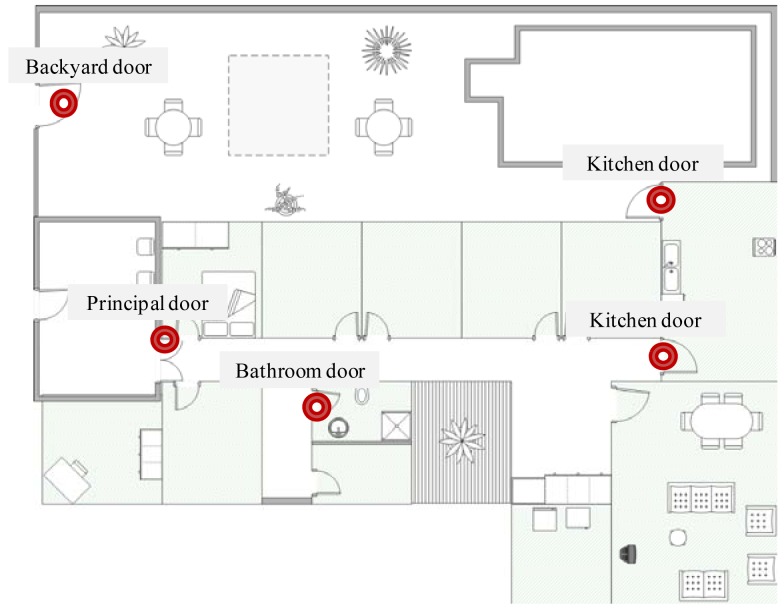
Map of the Nursing Home showing the location of RFID readers.

To monitor the ‘Entering into sensitive areas’ and ‘Escape from the house’ situations of care, RFID readers were installed on the kitchen and bathroom doors, as well as on the principal and patio doors of the residence (see Figure 7). We used an RFID system that works on 13.56 MHz with passive tags, so that they have to operate directly from the RF output of the RFID reader and the tags can be read 30 cm away from the reader. As mentioned above, each elder carried two RFID tags, which are identified (at least one of them) when passing through any of the instrumented doors. These situations of care are identified whenever an RFID tag referring to an elder is detected at a door, and no RFID tag referring to a caregiver is detected (we assume that in this case the older adult is passing through the door all alone).

**Figure 8.**
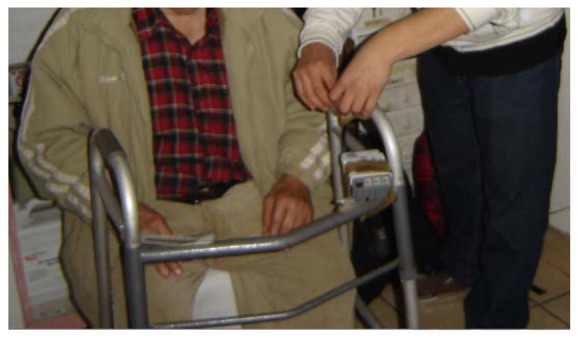
Installation of the pressure sensors on the handlebars of the elder's walking aid for the identification of the ‘Get up without assistance’ situation of care.

In the case of the ‘Get up without assistance’ situation of care, pressure sensors were installed in the seat of the chair of the elder and on the handle of the walking aid (see Figure 8), so that when the elder gets up, the sensor from the seat of the chair changes its status from 1 to 0 (pressure to no-pressure) and the sensor from the handle of the walking aid from 0 to 1 (no-pressure to pressure) when holding the handlebars.

**Figure 9.**
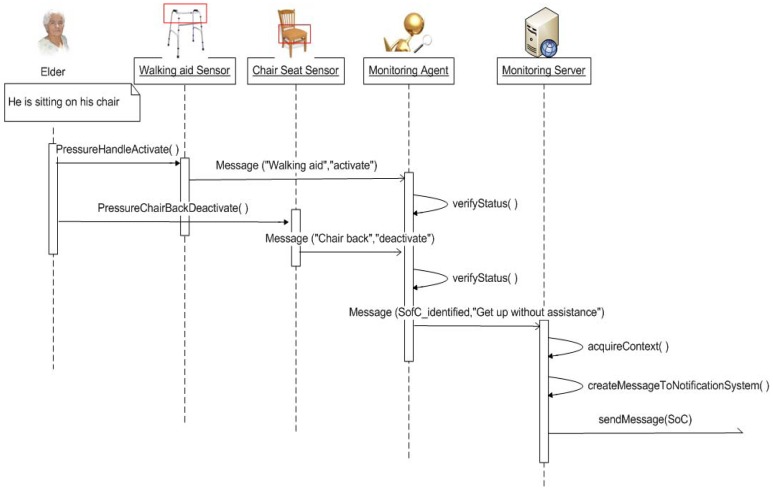
Sequence diagram for the inference of the ‘Get up without assistance’ situation of care.

Figure 9 shows a sequence diagram to illustrate the functionality of the complete monitoring system for the inference of a situation of care. The sequence starts with an elder sitting on a chair, with the sensor in the seat of the chair activated (1) and the sensor in the handlebar of the walking aid deactivated (0). When the elder gets up, the sensors send the change on their statuses to the Monitoring Agent in the system, so that it could verify the status of the elder and infer whether the situation of care has occurred. Once the situation of care is inferred, a message is sent to the Monitoring Server for its transmission to the Notification System.

The monitoring system implemented, albeit with some limitations, allows to detect and monitor various situations of care in the nursing home (e.g., Entering into sensitive areas, Escape from the house and Get up without assistance), demonstrating the feasibility of automatically identifying and inferring the situations of care that older adults and their caregivers face in their daily lives in the residence. The next two sections depict the development of two notification systems based on the CANoE notification model, which depend on a monitoring system, like the one presented in this section, for the acquisition of context.

## 4. Case Study 1: Context-Aware Notifications to Increase Awareness

As mentioned before, there are times at which caregivers cannot be aware of the situations of care that elders face in a nursing home. In these cases, it is necessary to introduce the use of mechanisms that provide support to solve this problem. We propose the use of context-aware notifications as a means to increase the awareness of caregivers about these situations of care, and as a way to improve their timely provision of care for the elderly.

The aim of this case study is to introduce a CANoE Notification System to support the care of elders with cognitive decline at nursing homes. We refer to this system as the CANoE-Aw Notification System. The scope of this system consists of providing notifications to the caregivers; that is providing them with messages, regarding the situations of care that arise at the nursing home.

Based on a characterization of the care giving process at a nursing home [12], we identified a set of requirements and insights to inform the design of the CANoE-Aw notification system. A further description of the system, of its implementation, and of the main results from an in-situ evaluation, is provided next.

### 4.1. Design of the CANoE-Aw Notification System

**Figure 10.**
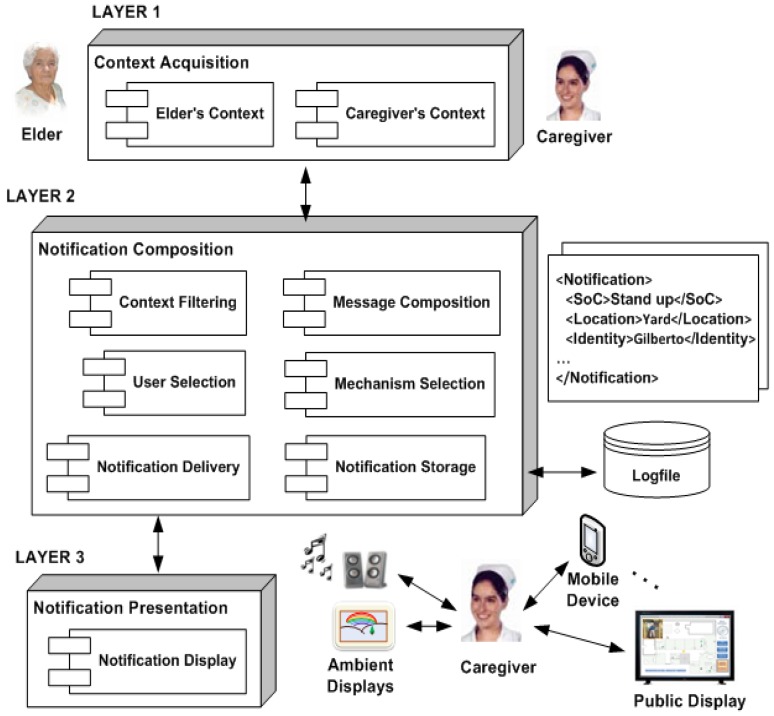
Architecture of the proposed CANoE-Aw System.

The development of the CANoE-Aw notification system is based on the architecture shown in Figure 10. Layer 1 is responsible for the communication with the monitoring systems for context acquisition (as described in Section 3). Layer 2 is the notification server, which contains an ensemble of modules that provide the services to adapt notifications. Lastly, layer 3 includes the notification mechanisms responsible for the presentation of information to caregivers.

Based on this architecture, we proposed the design of four notification mechanisms (also based on the ‘Design/Selection of Notification Mechanisms’ sub-model, described in Section 2). These mechanisms are further described next. The implementation details of the notification server are shown in the following sub-section.

The main design features of the CANoE-Aw notification mechanisms are described in Table 1.

#### Digital Portrait

**Figure 11.**
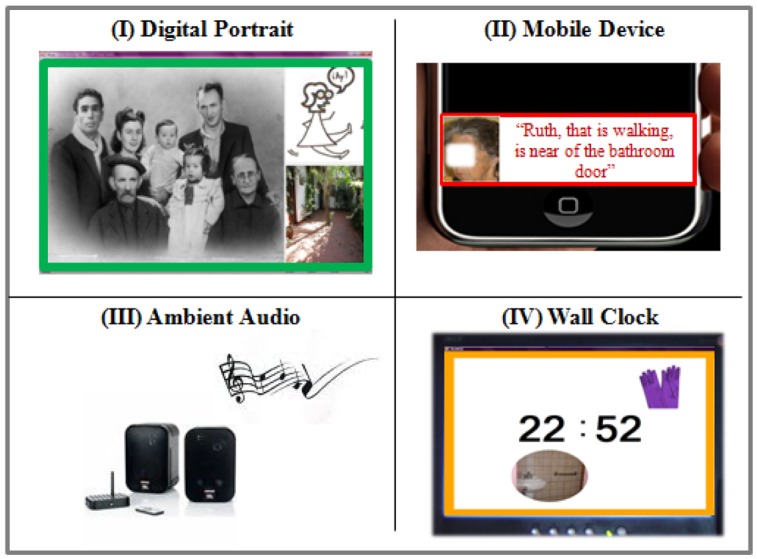
Implemented CANoE-Aw Notification Mechanisms.

This mechanism is an ambient device that visually displays the information on a public display, as shown in Figure 11(I). To capture the caregiver's attention it emits an auditory clue. The notification message is formed using three contextual elements: the elder's *Identity* using an elder's family photo; the *Situation of Care* by means of a sketch; and the *Location* of the situation of care by means of a photo of the place where the situation of care is occurring. The response priority is shown by means of the color of the frame (Red–High Priority, Yellow–Medium Priority, and Green–Low Priority). This mechanism was implemented using a computer monitor connected to a client-computer. Communication with the notification server was achieved via Wi-Fi.

#### Mobile Device

This mechanism is a portable device that visually displays the information, as shown in Figure 11(II). To capture the caregiver's attention it emits an auditory clue. The notification message can be formed using up to five contextual elements: *Identity, Situation of Care, Location, Activity* and *Time*. This mechanism duplicates the presentation of the Identity element using an elder's picture. The response priority is shown by means of the color of the frame. This mechanism was implemented on a touch PDA (HP iPAQ 610/610c) with a Wi-Fi connection.

#### Ambient Audio

This mechanism is an ambient device that displays the information as an auditory clue by means of a pair of speakers, as shown in Figure 11(III). The notification message is a sound track composed by two sounds: (i) the first sound represents the *Location* where the situation of care occurred, each location has associated a specific sound; and (ii) the second sound represents the response priority by means of an alarm tone, one for each urgency level. This mechanism was implemented using a laptop with wall-mounted speakers.

#### Wall Clock

This mechanism is an ambient device that visually displays the information using a device that could be already placed in the environment, as shown in Figure 11(IV). To capture the caregiver's attention it emits an auditory clue. The notification message can be formed using two contextual elements: the elder's *Identity* by means of an image associated to him (e.g., a pair of gloves), and the *Location* of the situation of care by means of a picture of the place. The response priority is shown by means of the color of the frame. This mechanism was implemented on a laptop with wall-mounted secondary display and speakers.

### 4.2. Implementation of the CANoE-Aw Notification System

The purpose of the notification server is to create and send notifications to all notification mechanisms in order to present the awareness information. The communication process between the server agents and mechanism agents was implemented using the XMPP instant messenger protocol through the Openfire server software [32]. Although evidence in the literature points to the existence of standard protocols for message exchanges in alerting systems, such as the *Common Alerting Protocol* [33], we decided to use a simpler and personalized message format using a more general message exchange protocol (*i.e.*, XMPP). As a future line of work, we propose to consider the use of CAP for the next version of the system.

The process for creating a CANoE Notification consists of three steps: Configure the notification contents (message adaptation), Assign response priorities to the caregiver (response priority adaptation), and Adapt the presentation of the notification to the caregiver (mechanism selection). A description of these steps, which follows the CANoE design guidelines, is provided next:

#### Step 1: Configuration of the notification content

**Figure 12.**
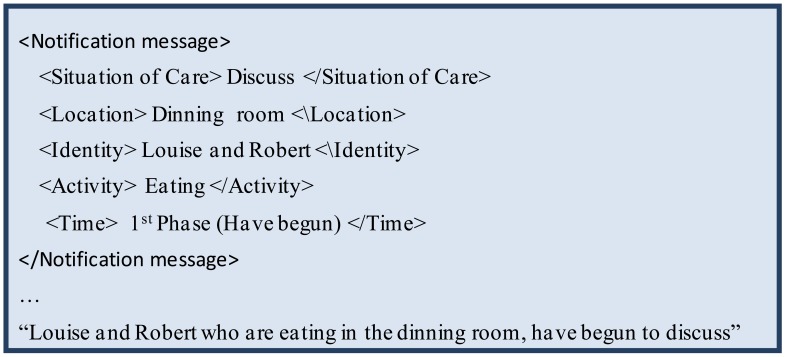
An example of an XML notification message.

This configuration is based on the “Composition of the Message” model presented in Section 2 (see Figure 2). According to the situations of care that arise at a nursing home [12], we defined two kinds of messages:

1. For Situations of Care in which elders could be at risk based on their location, *i.e.*, when an elder tries to ‘Escape from the nursing home’ (e.g., the main door and yard doors are open), and when an elder tries to ‘Enter into restricted areas’ (e.g., restroom and kitchen). In this case the message includes two contextual elements: Identity and Location.
2. For the remaining situations of care (e.g., ‘Discussions between elders’, ‘Get up and walk without assistance’, *etc.*). In these cases the message includes from three to five contextual elements: Situation of Care, Identity, Location, Activity and Time. Figure 12 shows an example of this kind of message.

#### Step 2. Assign response priorities to the caregiver

**Figure 13.**
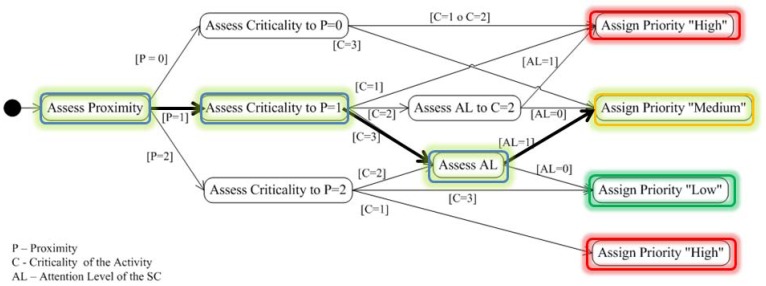
Decision tree to establish the response priority of the caregiver.

Whenever a situation of care arises, a response priority is assigned to each caregiver in order to help them in their coordination process. This assignment is based on the “Configuration of Response Priority” sub-model presented in Section 2 (see Figure 3). The priority is defined based on the three dimensions of the model: (i) Attention Level for the situation of care; (ii) Proximity from the Caregiver to where the situation of care is taking place; and (iii) Attention Availability of the caregiver (obtained by means of the criticality level of the caregiver's activity). Later, these values are used as inputs for a decision tree in order to define the response priority (see Figure 13). Finally, the system verifies that a “high” priority had been assigned to at least one of the caregivers.

Figure 13 illustrates how the response priority of a caregiver is assigned using a decision tree. The path is highlighted using bold arrows and colored round boxes: First, the caregiver's **proximity** to the situation of care is evaluated, if it is “Near”, the assigned value is “1”; later, the caregiver's availability is evaluated by taking into account the **criticality of the activity** that s/he is performing at that moment. If the availability is “High” (e.g., elder's hygiene activity), the value is “3”; and finally, the **attention level** related to the situation of care is evaluated. If this level is “Immediate”, the assigned value is “1”. Thus, the response priority of the caregiver can be assigned as ‘*Medium*’. The procedure is executed for each caregiver and when all values are computed, a sorting process is conducted in order to ensure that a ‘High’ priority is assigned to at least one caregiver.

When there is a notification that is left unattended, as time passes (e.g., 30 seconds), the response priority required to each caregiver will increase. This may occur in two cases. The former when there is a change from a lower urgency level situation of care, to a higher urgency level situation of care (e.g., from “close to the door” to “entering into a sensitive area”). The latter after every notification repetition because the unattended notification will automatically generate an increase in the response priority level required for all caregivers.

#### Step 3. Adapt the presentation of the notification to the caregiver

This adaptation is based on the “Design/Select Notification Mechanism” sub-model presented in Section 2 (see Figure 4). To select the mechanism, it is necessary to evaluate the notification's requirements based on the mechanism's features previously designed for the system. The CANoE-Aw notification system consistently considers notifying situation of care with the following default configuration levels: the attention level is “Immediate”; the intrusion level is “Disrupt”. These default values were due given the environment features established on the design process. Hence, two values are defined on-the-fly by the system: (i) the Amount of required context of the situation of care; and (ii) the Available Perception Channels of the caregiver. These values are used as inputs for the decision tree in order to select the mechanism with which to notify to each caregiver.

**Figure 14.**
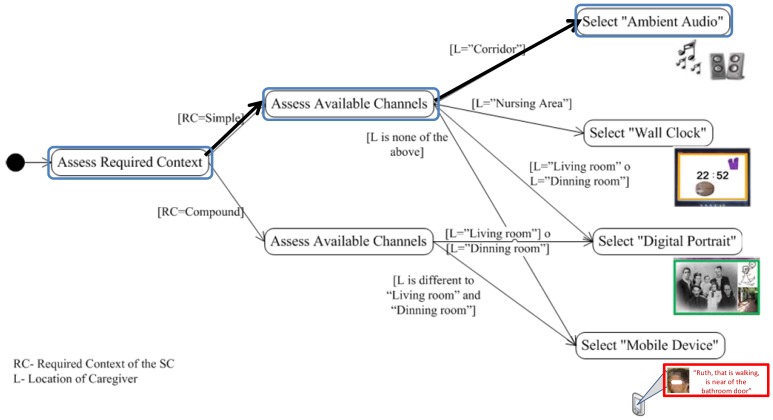
Decision tree to select the notification mechanism.

Figure 14 illustrates how the presentation mechanism is selected by the CANoE-Aw notification system using a decision tree. The path is highlighted using bold arrows and colored round boxes: First, the **required context** of the situation of care is evaluated, if it is ‘simple’, then, the **available channels** of the caregiver are evaluated based on his/her location. In this case the location is the ‘corridor’, and it is thus assumed that his/her auditory channel would be available; therefore the ‘*Ambient Audio*’ mechanism is assigned. The auditory notification is created aggregating a sound associated to the elder's location (e.g., kitchen–the sound of kitchen tools that fall to the floor) and a sound that corresponds to the response priority of the caregiver (e.g., using a high pitch alarm tone).

### 4.3. Evaluation

The evaluation study was conducted at the same nursing home where the characterization was performed. The evaluation was conducted during 21 days. During this time, 12 elders were living there, seven of them with cognitive impairment. The purpose of the evaluation was to obtain the caregivers' perception regarding CANoE-Aw notifications. Their perception is related to: (i) knowing whether the notification was correctly received; (ii) knowing whether the notification's context was correctly interpreted, and the notified situation of care was correctly inferred; and (iii) knowing whether the response priority was perceived and correctly interpreted.

#### 4.3.1. Context of the Study

Five caregivers participated in the evaluation, one female and four male. They had an average age of 21 years old. During the notification period, and according to the shift rotation schedule, two caregivers worked during three morning shifts (from 7 am to 3 pm). One of the caregivers participated in two shifts. They performed their everyday care activities, along with the cleaning and information management activities of the nursing home.

**Figure 15.**
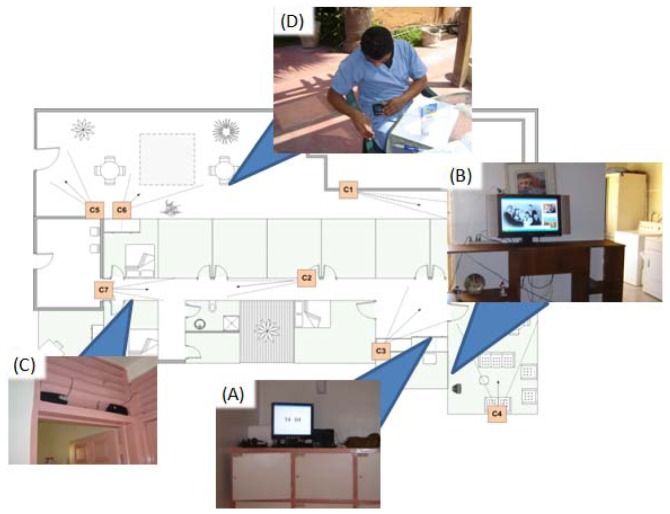
*CANoE-Aw notification mechanisms* installed at the nursing home: (**A**) ‘Wall Clock’ at the Nursing Area; (**B**) ‘Digital Portrait’ at the Dinning-Living room; (**C**) ‘Ambient Audio’ at the Corridor; and (**D**)‘Mobile Device’ carried out by each caregiver.

The CANoE-Aw notification system was installed and configured in the nursing home as shown in Figure 15. Previous to the evaluation, we conducted preliminary tests and several pilot sessions where caregivers used the notification mechanisms for the first time.

Although we had access to a basic infrastructure to perform the participants' monitoring (See Section 3), we were not able, nor given permission, to install the required sensors for the monitoring of the 12 elders and the 2 caregivers at the actual site. Thus, to capture the elders' and caregivers' context we used the Wizard of Oz technique [34], by following elders and caregivers through an *ad-hoc* close-circuit video infrastructure.

During the seven days of the study, CANoE-Aw notifications were sent to caregivers whenever situations of care occur. For every notification sent, caregivers were asked to answer a questionnaire in order to evaluate his/her perception regarding the notification. At the end of this study, participants were interviewed in order to gather qualitative information about their perception.

#### 4.3.2. Evaluation Results

Within the notification period, 48 situations of care were identified, which generated 96 notifications that were sent to the caregivers (two notifications—one to each caregiver—by each identified situation of care). A description of the impact of the notifications at the nursing home, and of the caregivers' perception about their use, are provided next.

##### Impact of the CANoE-Aw Notifications

The results from the observation and from on-exit questionnaires provide evidence that caregivers, in most cases, were not aware of the situation of care, until it was notified (63.08%). This explains why when caregivers were asked about it, they answered that they perceived an increase in their awareness of the situations of care.

During the interviews, caregivers stated that notifications were very useful, and that timing is very important in the elderly care process. One of them stated it this way: “*if the notification had not been sent nor correctly perceived [by the caregiver], the situation [of care] would have turned into a very dangerous one*” (Caregiver 2).

Table 2 shows some of the quantitative results regarding perceived notifications, and correct context and response priority interpretation regarding each proposed mechanism (first column). Firstly, we show the amount of notifications perceived by caregivers per mechanism (*n* = 78, second column). As mentioned in the CANoE-Aw implementation section, the notification mechanism is defined based on the current situation of the caregiver (activity + location). Then, the third column presents the amount of these perceived notifications for which all the contextual elements were correctly interpreted. Finally, the fourth column presents the amount of the perceived notifications for which the response priority was correctly interpreted. As an example, consider the case of the Digital Portrait mechanism (second row), for which caregivers perceived a total of 16 notifications, from which they correctly interpreted all the contextual elements for 15 of them (93.75%), and from which they correctly interpreted the response priority for 14 of them (87.5%).

##### Validation of the CANoE Notification Model

The use evaluation of the CANoE-Aw notification system, allowed us to validate the design guidelines of the CANoE notification model. A description of the main results is provided next:

- **Configuration of the Content of the Notification.** Caregivers considered useful setting the notification message according to the context of the Situation of Care. Quantitatively, from the total number of notifications received by caregivers (*n* = 78), 96.16% of these were correctly interpreted based on the notification message. Qualitatively, caregivers expressed that in the situations of care where the elderly were at risk due to their location, just becoming aware of that location through the notification message was enough information as to make the decision and go to provide the required care. This in contrast to other situations of care, where it was necessary to know all the contextual elements. One of the caregivers put it this way: “*It was important to know what activity [the elderly] was doing* (*situation of care*), *apart from where it was happening* (*location*), *as being in his room, you think everything is all right and that he is just asking for a snack or something*” (Caregiver 1).
- **Assign Response Priority to Caregiver.** In general terms, caregivers' perceived the use of the response priority of the notification as useful. 80% of the caregivers (4/5) reported that the response priority was useful when they have to make a decision: “*The response priority indicates whether you are the most available caregiver to attend the situation at that moment, or whether you are the busiest one* … *so that you can decide if you have to go* … *or if your partner has to go*” (Caregiver 5). Additionally, caregivers perceived that the response priority allowed them to achieve a better coordination regarding the attention of a situation of care. In this case, the following behaviors were observed: (i) there were occasions when the caregiver received a ‘Medium’ priority response and s/he was performing an activity with a High criticality level; in this cases, the caregiver decided to continue performing his activity; and (ii) in other occasions (3.8% of all notifications) the caregivers did not paid attention to the response priority and went to attend the situation of care because they knew that their condition was ‘Available’. This was due to the Low criticality of their activity, thus the information sent by the system did not affect his/her perception, or his ability to make the decision.
- **Adapt Presentation to Caregiver.** Caregivers considered useful being notified through appropriate mechanism that took into account their activity and location. Two examples that illustrate the aforementioned include: (i) whenever the caregiver performed the moving activity of an elder through the corridor (e.g., using a wheelchair) notifications were presented to the caregiver using ambient audio, which could be perceived without interrupting the moving activity. In this case the caregiver stated that: “*I did not have to look at the notification [mechanism], thus, it was better to listen to it*” (Caregiver 1); and (ii) when the caregiver was in the nursing area performing a communication activity (*i.e.*, in a phone call), a visual notification arrived, and the caregiver stated that: “*if you are on the phone you just have to turn and see the notification, and thus realize what the situation is*” (Caregiver 5). Caregivers indicated that almost all notifications were appropriated, based on their location and current activity at that moment. On the one hand, the notifications from the three ambient mechanisms (*n* = 40) were perceived 100% by caregivers. On the other hand, there were limitations in some of the implemented mechanisms. For instance, the mobile device did not cover this adaptation 100%, as it only implemented auditory cues and in some cases (20.4%, *n* = 49) the caregiver did not hear the notification because he was in a noise environment.

## 5. Case Study 2: Context-Aware Notifications to Support the Coordination of Caregivers

As mentioned before, in some care settings such as nursing homes, the tasks performed by an individual caregiver are part of collective activities performed by a group. They are not only individual work, but also work with input from others and in consensus with others. For this reason, systems that support collaborative work play an important role in this type of environments. According to [35], effective communication and coordination can be enhanced if group activities are coordinated with the help of technology. An example of this is *CareTwitter* [36], where the members of a caregiver network coordinate themselves based on contextual information about the care activities, locations and times.

The lack of this kind of knowledge may get groups into conflict, and could cause that important care actions be repeated or skipped. Furthermore, coordination support should not be restricted to the typical daily activities of the caregivers, but also to include support during care emergency activities.

The aim of this case study was to design and develop the CU-IDA system, in order to facilitate the coordination process among caregivers while they address a situation of care that has been notified. Its development is based on the CANoE Notification Model, described in Section 2, and relies on the existence of the Monitoring and the CANoE-Aw Systems (which were shown in Sections 3 and 4, respectively). The following sections describe the design of a communication-coordination process for caregivers, the implementation of the CU-IDA system and the main results of a preliminary evaluation.

### 5.1. Design of a Communication-Coordination Process

We propose a communication-coordination process to support caregivers in their attending the situations of care, providing them with valuable knowledge about the current status of each caregiver. The process consists of the following three phases:

**Phase 1.** Provide caregivers with awareness about the status of each one of their peers (Caregiver Situation [12]), this state is composed of the following contextual information: caregiver's identity, current activity and location, identity of the elder that the caregiver is currently attending, and the availability of the caregiver with respect to the rest of their peers.
**Phase 2.** Capture the decision of a caregiver who decides to attend the elder's situation of care. If more than one caregiver makes this decision, the system captures the first incoming decision and notifies other caregivers about it. The additional decisions are also captured. If after 30 seconds of the notification there is no answer from a caregiver, the system restarts from Phase 1.
**Phase 3.** Notify caregivers when someone has taken the decision to “attend” the situation of care, providing the identity of the caregiver.

The first and third phases correspond to a notification mechanism, in the first case to inform regarding the context of care givers, and in the second case to inform about the decision to “respond” by one of the caregivers. We decided to establish a communication architecture that could support this process and various context-aware communication mechanisms. Two communication mechanisms were designed: (i) a *visual-tactile mechanism*; and (ii) an *auditory-tactile mechanism*. The former presents information to caregivers in a visual manner, which is considered as a non-intrusive presentation method. The latter proposes the use of audio to present information, in order not to interfere with the visual channel of caregivers while they perform their activities.

Table 3 shows the design features of each notification mechanism used in Phases 1 and 3 of the process described above. The design of the mechanisms was based on the Model for the ‘Design and Selection of the notification mechanism’ presented in Section 2.

**Figure 16.**
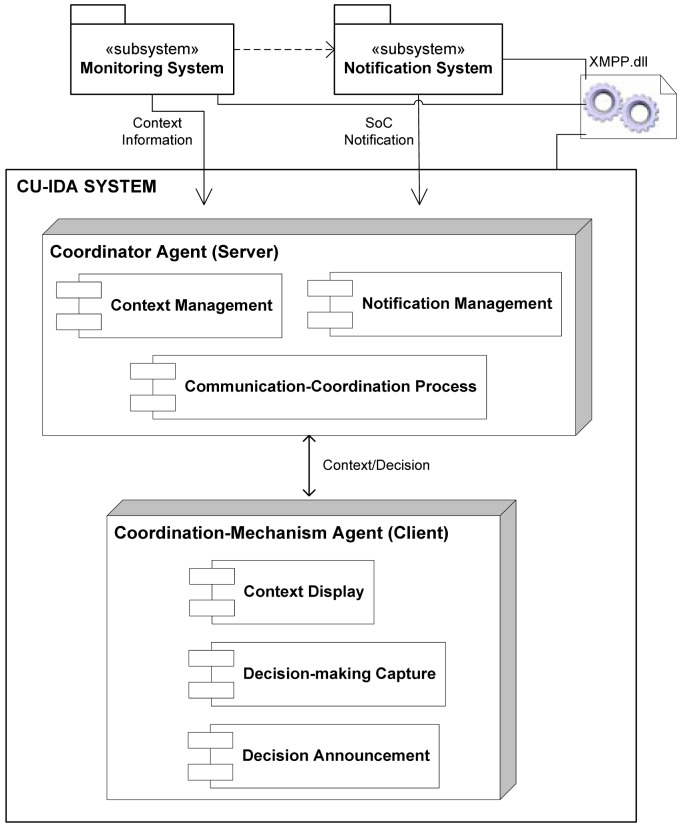
Architecture of the CU-IDA System.

The construction of the CU-IDA system, an extension of the CANoE-Aw system used in case study 1, was based on the proposed architecture shown in Figure 16. It is a client-server architecture using multiple agents. A “Coordinator” agent acts as a server agent and several “Coordination Mechanism” agents act as clients. The main functions of the Coordinator agent include communication with the monitoring and the notification systems to carry out the communication-coordination process.

### 5.2. Implementation of the CU-IDA System

**Figure 17.**
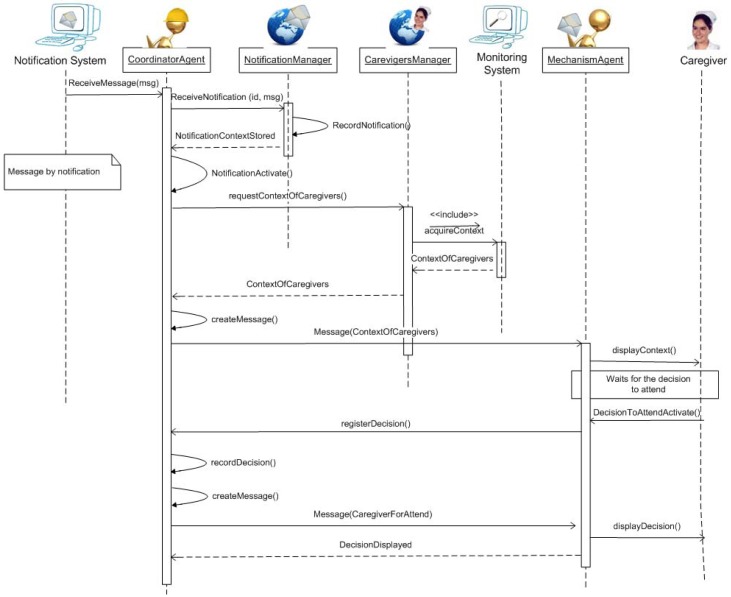
Sequence Diagram of the Communication-Coordination Process.

The communication-coordination process starts when the Coordinator agent is notified by the Notification System that a Situation of Care has occurred. Then, the coordinator agent stores the notification and retrieves the context of the caregivers from the Monitoring System. Subsequently, this information is sent to each caregiver by means of the portable or ambient mechanisms. Finally, the coordination mechanism agent waits for the decision from the caregiver that will attend the situation of care, which is then announced to the other caregivers. This process is described in the sequence diagram presented in Figure 17. Communication with these systems, as well as with the Coordination mechanism agents are supported by means of the XMPP protocol using the Openfire server software [32]. The following lines provide details of the mechanisms implemented.

#### 5.2.1. Visual-Tactile Mechanism (PortaCC)

**Figure 18.**
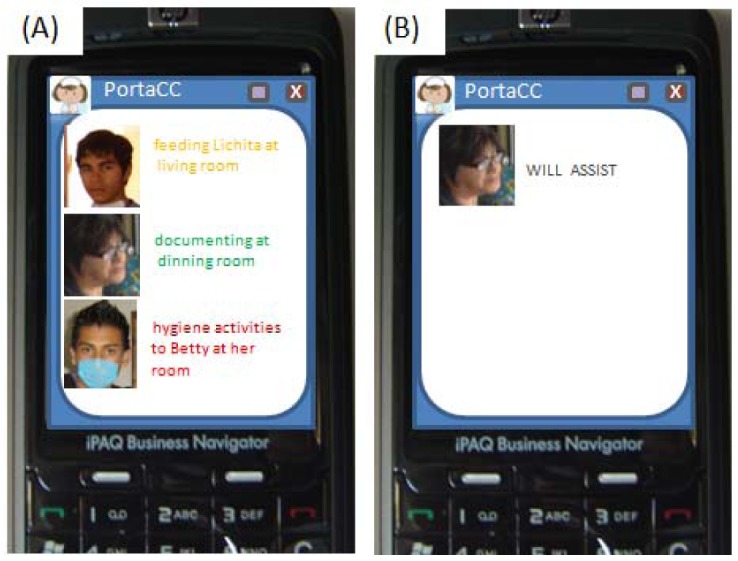
Visual-Tactile Mechanism Notifications: (**A**) Phase 1: Caregivers' Context; (**B**) Phase 2: Decision Announcement.

The visual-tactile mechanism is implemented on a mobile device (Touch PDA). We used an HP iPAQ 610/610c Smartphone. The application was implemented in C#, using sockets for the communication with the coordinator agent (server). The functionality provided by this mechanism is illustrated next. Firstly, the caregiver is authenticated, and then the coordination mechanism agent (client) waits for a message from the coordinator agent (server) that indicates the beginning of the communication process. Once this process is started, the mechanism is notified about the context of the involved caregivers (Figure 18 (A)). Also, the caregiver with the highest availability may touch his picture on the PDA screen to indicate that s/he will attend the situation of care. This decision is subsequently sent and shown to the other caregivers (Figure 18(B)), thus ending the communication process.

#### 5.2.2. Auditory-Tactile Mechanism

This mechanism consists of three devices: a Smartphone with headset (Personal Audio), a touch sensor (smart button) and a pair of speakers (ambient audio). This mechanism was implemented using a Nokia N95 Smartphone with Wi-Fi connectivity. The application was implemented in Java ME.

The main feature of this mechanism is that it only presents information on the user's availability with respect to their peers (*i.e.*, present a single contextual variable). This is done in the first phase of the communication process through three different alarm tones associated with the three levels of availability (*i.e.*, Tone 1 meaning High availability, Tone 2 meaning Medium availability and Tone 3 meaning Low availability).

**Figure 19.**
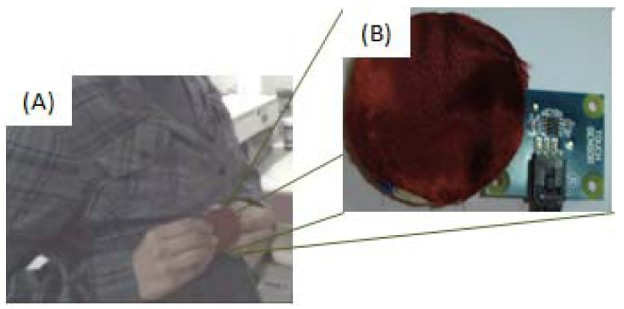
Smart button used to capture the caregiver's decision (**A**), implemented using a touch sensor (**B**).

In the second phase, the caregiver can indicate the decision to attend the situation of care through the smart button s/he is wearing (see Figure 19(A)). The button consists of a Phidget 1100 touch sensor (see Figure 19(B)). This application was programmed in C#.

Finally, to complete the communication process a sound that represents the identity of the caregiver who will attend the situation of care is sent as a notification. This is sent either via the Smartphone (personal audio) or via the speakers (ambient audio) according to the caregiver's context (e.g., location). Each caregiver is represented by a specific sound, which is learned by the other caregivers. This design decision was inspired by the Earcons concept [37]. Further, we propose to use ambient audio notifications for caregivers that are performing activities that do not allow them to use the portable smart button, such as when they are bathing an elder.

### 5.3. Qualitative Evaluation

Following the implementation of these mechanisms, we conducted a scenario-based qualitative evaluation designed to gather caregivers' perceptions regarding the impact of the use of the context-aware communication mechanisms in the nursing home. The aim of this type of evaluation was to obtain feedback from actual users during projected use to inform the re-design of an application [38].

#### 5.3.1. Use Scenarios

We prepared two videos depicting projected scenarios of use of the implemented tool, each one illustrating the use of one of the two mechanisms (visual-tactile and auditory-tactile), respectively. These scenarios specify: (i) a situation of care in progress; (ii) the notification of this situation of care to caregivers; and (iii) the use of the corresponding mechanism to coordinate the provision of care for the reported situation of care.

#### 5.3.2. Context of the Evaluation Study

##### Goal

Obtain the perception of caregivers regarding the usefulness and ease of use of the proposed communication-coordination system, including that of the proposed mechanisms.

##### Participants

Five caregivers from a nursing home participated in the study. Participants from the different shifts (morning, afternoon and evening) were included.

##### Procedure

Firstly, we demonstrated the overall system functionality, including that of the two proposed mechanisms. Later, we allowed caregivers to become familiar with the use of each of the proposed mechanisms. Then, we conducted a focus group, where the videos of projected scenarios of use corresponding to each mechanism were shown. After the presentation of each video, participants were asked to fill out a questionnaire (Likert scale of 5), with respect to the perceived usefulness and ease of use of the mechanisms proposed.

#### 5.3.3. Evaluation Results

Most caregivers (four out of five) indicated that having a communication system greatly benefits them in their work. In addition, all caregivers are willing to use both communication-coordination mechanisms. With regard to the usefulness and ease of use, the mechanism that they found most useful (average of five) was the visual-tactile mechanism (PDA), although it was also rated as the least easy to use (2.5).

Conversely, the auditory-tactile mechanism received a lower qualification regarding its usefulness (three out of five), but caregivers perceived it as the easiest to use (4.3). A possible explanation for this could be that caregivers receive information via an audio channel without having to perform any further action (*i.e.*, they do not need to change their visual focus). On the other hand, the low rating of this mechanism regarding usefulness could be due to the lack of richer contextual information mechanisms, as it only shows the availability of the caregiver relative to that of their peers).

In general, most caregivers (four out of five) considered that the use of the mechanisms would improve their process of care for the elderly. In addition, providing awareness about the activities of the group of caregivers helps them making a decision regarding who has to attend the notified situation of care: “*I have to know what my colleagues are doing, if they are performing more activities, or if those activities are more critical than those that I am performing*” (Caregiver 4). Further, getting to know the identity of the elder that their colleagues are attending allows caregivers to evaluate their availability: “*Yes, getting to know the activity is important, but also, knowing whom they are attending is very important too; that is, if they are with* <*Elder 4*> *they can not leave her just like that, and I know instantaneously that she is busier that me*”.

Caregivers perceived a potential benefit in the use of the proposed technology, and said that they would be interested in having it installed in the nursing home to support their work routines. They also perceived that the benefit would be both for caregivers and elders: “*Thanks to this technology we are able to be more attentive for the provision of care to the elderly*” (Caregiver 1).

## 6. Conclusions and Future Work

The main contribution of this work is the CANoE notification model, which is a tool to develop context-aware notification systems for critical environments, such as nursing homes. This model considers adapting the notifications based on three contextual source entities (the environment, elders and caregivers). To do so, it proposes three design guidelines: (i) Configure the notification's content; (ii) Assign response priorities to caregivers; and (iii) Adapt the presentation of the notification.

Other research works have considered adapting the notifications (in an *active* manner) [18,19], but these works do not consider the physical availability of the notification receiver in order to adapt the notification presentation. Our proposal, by means of the three design guidelines, considers as a principle to adapt the presentation of the notification in an active manner, not only based on the issuer's context (elder), but also on the receiver's context (caregiver). The aim of this principle is try to ensure that caregivers perceive and understand the notification.

The results of the two case studies provide evidence of the feasibility and applicability of the CANoE notification model. The first case study was proposed as a notification solution for caregivers in a nursing home with the aim of increasing their awareness about the elders' situations of care. The second case study was proposed as a solution to provide caregivers with a mechanism to support their coordination process to address a situation of care whenever it arises. This mechanism aims at providing a follow up to the notifications sent, so that the notified situation of care could be attended. The results from the first case study reveal that in a critical environment, as a nursing home, it is essential to take into account the context of the notification receiver and of the environment to adapt the notifications. Although, participant caregivers perceived the implemented mechanisms as useful and ease to use, other aspects must be revised (e.g., to provide a tactile-vibratory-cue in addition to the auditory cue), in order to provide a more comprehensive scope towards the situations that a caregiver may face. Regarding the results of the second case study, caregivers perceived that: (i) the use of the proposed communication-coordination mechanisms (based on the CANoE notification model) would improve the care process of elders in a nursing home; (ii) the proposed coordination mechanisms would allow them to know who would attend the situations of care; and (iii) that the proposed mechanisms are useful and ease to use.

Directions of future work are three-fold. Firstly, we are considering conducting an additional case study, in order to evaluate the CANoE notification model from the perspective of system developers; and this with the aim of expanding the design guidelines to make them more prescriptive, while making them more flexible and adaptable to the features of other critical environments, in addition to those of nursing homes. Secondly, with regard to monitoring systems, we propose to extend the proposed basic architecture, so that it could integrate existing monitoring technologies for context acquisition. We will also work on further developing and validating the basic context acquisition mechanism with the aim of making it more comprehensive regarding the situations of care of interest, and measuring its performance and accuracy, in order to achieve a more reliable, effective and low-cost solution. A possible approach is based on additional situations of care in which the inference process could be improved. For instance, Table 4 describes some of these situations and some schemes that could be used to address them based on current research reported in the literature. Finally, a third direction of future work regards to a comprehensive evaluation of the proposed systems, which render information about the performance and accuracy of the context acquisition and inference mechanisms, as well as about actual use of the system in the nursing home.

## Figures and Tables

**Table 1.** Design features of the CANoE-Aw Notification Mechanisms implemented.

**Mechanism**	**Attention Level**	**Amount of required context**	**Perception channels**	**Intrusiveness Level**

**Digital Portrait**	Immediate	Compound: three contextual elements (I, SC, L)	Visual Auditory Clue	Disrupt
**Mobile Device**	Immediate	Compound: five contextual elements (I, SC, L, A, T)	Visual Auditory Clue	Disrupt
**Ambient Audio**	Immediate	Simple: one contextual element (L)	Auditory	Disrupt
**Wall Clock**	Immediate	Compound: two contextual elements(I, L)	Visual Auditory Clue	Disrupt

Contextual elements: I—Identity, SC—Situation of Care, L—Location, A—Activity, T—Time

**Table 2.** Quantitative evaluation results for the CANoE-Aw system.

**Mechanism**	**Perceived Notifications**	**Notification Context correctly interpreted**	**Response Priority correctly interpreted**

Wall Clock	13 (16.66%)	13 (100%)	10 (76.92%)
Digital Portrait	16 (20.52%)	15 (93.75%)	14 (87.5%)
Ambient Audio	10 (12.82%)	10 (100%)	10 (100%)
Mobile Device	39 (50.00%)	37 (94.87%)	36 (92.31%)

**TOTAL**	**78 (100%)**	**75 (96.16%)**	**70 (89.74%)**

**Table 3.** Design characteristics of the CANoE notification mechanisms used in this case study.

**Mechanism**	**Attention Level**	**Amount of required context**	**Perception channels**	**Intrusiveness Level**
**Visual-Tactile** **(Phase 1)**	Immediate	Compound: 4 contextual elements (I, L, A, Av)	Visual Auditory Clue	Disrupt
**Visual-Tactile** **(Phase 3)**	Immediate	Simple: single contextual element (I)	Visual Auditory Clue	Disrupt
**Auditory-Tactile** **(Phase 1)**	Immediate	Simple: single contextual element (D)	Auditory	Disrupt
**Auditory-Tactile** **(Phase 3)**	Immediate	Simple: single contextual element (I)	Auditory	Disrupt

I—Identity, L—Location, A—Activity, Av—Availability

**Table 4.** Schemes proposed to infer complicated situations of care.

**Situation of Care**	**Schema**	**Method proposal**

Discussions and aggressive behavior between patients	Shouting Aggression between people	Audio sensors [39] Computer vision [40]
Aggressive behavior towards caregivers	Aggression between people	Computer vision [40]
Help to stand up or walk provided by another patient	Motion of the wheelchair	Motion sensors [22,41]
